# Multidimensional Profiling of Chinese Sweet Tea *(Lithocarpus litseifolius)*: Processing Methods Modulate Sensory Properties, Bioaccessibility and Prebiotic Potential via Gut Microbiota Regulation

**DOI:** 10.3390/foods15010110

**Published:** 2025-12-30

**Authors:** Zhen Zeng, Qiyun Zhang, Lijia Zhang, Baichuan Hu, Xinyue Wen, Zihan Wang, Wenjuan Wu, Yuntao Liu

**Affiliations:** 1 College of Food Science, Sichuan Agricultural University, 46# Xinkang Road, Yaan 625014, China; 2 College of Science, Sichuan Agricultural University, Yaan 625014, China

**Keywords:** sweet tea, tea-making method, in vitro simulated digestion, in vitro fecal fermentation, antioxidant activity, prebiotic properties

## Abstract

This study systematically examines the effects of processing methods (green vs. black tea) and preparation techniques (brewing vs. decoction) on the flavor and functional composition of Chinese sweet tea (*Lithocarpus litseifolius*). Fermentation degree and extraction temperature were found to significantly influence polyphenol bioavailability, with green tea exhibiting the highest polyphenol and flavonoid contents (144.51 mg/g and 88.97 mg/g, respectively), while black tea showed an approximately 40% reduction in catechin levels due to oxidative polymerization. During in vitro simulated digestion, green tea maintained strong antioxidant activity despite its stronger bitter–astringent taste. Notably, in vitro fecal fermentation experiments demonstrated that sweet tea significantly promoted short-chain fatty acid (SCFA) production and modulated gut microbiota composition (with a 3.2-fold increase in acetate content in the black-tea decoction group). Black tea particularly enhanced beneficial genera (Roseburia and Coprococcus) after 24 h fermentation (*p* < 0.05) and exhibited superior prebiotic properties. Principal coordinate analysis confirmed there were significant structural differences in microbial communities among the treatment groups. This study is the first to reveal that processing methods regulate the prebiotic efficacy of sweet tea by modulating the bioaccessibility of active compounds, providing a theoretical foundation for the development of functional sweet tea products.

## 1. Introduction

*Lithocarpus litseifolius* (Hance) Chun, also known as sweet tea, belongs to the Fagaceae family and has been consumed for about 1600 years [1]. It was recognized as a new food-ingredient plant by the Chinese National Health and Family Planning Commission in 2017. Studies have demonstrated the excellent antioxidant capacity of sweet tea and its components (phlorizin, trilobatin, polysaccharides, and tea pigments) through both in vitro and in vivo experiments [2,3,4]. Beyond their influence on antioxidant activity, these components also modulate flavor profiles: catechin compounds within tea polyphenols predominantly contribute to the bitterness and astringency of tea, whereas dihydrochalcone derivatives are primarily responsible for its sweetness [5,6].

The bioactivity and flavor of sweet tea are influenced by processing. To preserve polyphenols and dihydrochalcones, tender leaves are often processed into unfermented teas like green tea. In contrast, black tea is a fully fermented tea, primarily containing tea polyphenols and oxidized catechin polymers (such as theaflavins, thearubigins, and theabrownins), amino acids, etc. [7]. Notably, during the fermentation process, the catechins are converted into complex polymers [8]. After fermentation, sweet tea’s dihydrochalcone and catechin levels drop significantly, with the dihydrochalcone content remaining higher than that of catechin [9].

Furthermore, the brewing method used significantly influences the leaching efficiency of tea components and the concentration of active ingredients. For example, brewing Rizhao Maojian (a famous green tea variety known for its tight, slender appearance and chestnut-like aroma that was obtained from local tea plantations in Shandong Province, China) using a higher tea-to-water ratio and temperature and for a greater duration raises the concentration of tea polyphenols and antioxidant activity in the tea [10]. A tea infusion brewed with distilled water for 30 min at 85 °C has the highest antioxidant capacity and total phenolic content for green tea leaves [11]. Moreover, prolonged high-temperature steeping potentially accelerates the degradation of residual pesticides in tea leaves [12]. An optimal water temperature and steeping duration are crucial for extracting tea components without causing oxidative decomposition of polyphenols and increasing bitterness and astringency.

Despite the long history of sweet-tea consumption, comprehensive studies on its fermented forms and the optimization of consumption methods for health benefits remain limited. Given the growing consumer focus on dietary health, this lack of scientific evidence may hinder market acceptance and commercial development. To address this knowledge gap, the present study systematically evaluates both unfermented and fully fermented sweet tea prepared through brewing and decoction methods, with a focus on their sensory characteristics, digestive behavior, and fermentation-induced functional properties.

## 2. Materials and Methods

### 2.1. Materials and Reagents

Sweet tea leaves (5–7 cm long) were collected in May 2023 from Lushan County, Ya’an City, Sichuan Province. Subsequently, the leaves were processed into sweet green tea and sweet black tea, respectively. Green tea is a non-fermented tea whose core principle lies in the high-temperature de-enzyming (kill-green) process, which rapidly inactivates polyphenol oxidase in fresh leaves and thus prevents enzymatic oxidation, thereby maximally preserving the tea’s natural components. After de-enzyming, the leaves undergo rolling for shaping and partial cell disruption to facilitate the infusion of internal compounds, followed by drying to fix the quality and develop aroma, resulting in the characteristic “clear liquor, green leaves, fresh and astringent” profile of green tea, with active components predominantly consisting of non-oxidized catechins [13]. Black tea is a fully fermented tea, and its quality hinges on withering to soften the leaves and enhance enzyme activity, followed by rolling to thoroughly disrupt cell structure, allowing polyphenols to make contact with polyphenol oxidase. Under controlled temperature and humidity conditions, fermentation enables intense enzymatic oxidation, generating characteristic components like theaflavins and thearubigins, and finally drying terminates fermentation, yielding the typical “red leaves, red liquor, mellow and sweet” quality of black tea [14].

Salivary amylase (3700 U/g), bile extract, and monosaccharide standards (ribose, fucose, galactose, glucose, galacturonic acid, glucuronic acid, arabinose xylose, rhamnose, and mannose (HPLC ≥ 98%)) were provided by Yuanye Biotechnology Co., Ltd. (Shanghai, China). Catechin compound standards ((+)-catechin (C), (−)-catechin gallate (CG), (−)-epicatechin (EC), (−)-epigallocatechin (EGC), (−)-epicatechin gallate (ECG), (−)-epigallocatechin gallate (EGCG), (−)-gallocatechin gallate (GCG), (+)-gallocatechin (GC), and gallic acid (GA)) (HPLC ≥ 98%) were purchased from Chengdu Lemetian Pharmaceutical Technology Co., Ltd. (Chengdu, China). Pepsin (3000 U/g) and pancreatin (4000 U/g) were purchased from Sangon Biotech Co., Ltd. (Shanghai, China). DPPH and ABTS reagents were procured from Sigma-Aldrich Corp. (St. Louis, MO, USA). Bile extract was provided by Yuanye Biotechnology Co., Ltd. (Shanghai, China). SCFAs (GC ≥ 99.5%) were obtained from Aladdin Biochemical Technology Co., Ltd. (Shanghai, China). All reagents and chemicals used were of analytical grade.

Prior to analysis, all instruments were calibrated. Daily verification of spectrophotometers was performed using standard references. HPLC/GC systems were calibrated for retention time and response factor prior to sample runs.

### 2.2. Experimental Methods

#### 2.2.1. Sweet-Tea Preparation

Two preparation methods, brewing and decocting, were used to prepare the sweet green tea and sweet black teas, resulting in four groups: green-tea brewing (GB), black-tea brewing (BB), green-tea decoction (GD), and black-tea decoction (BD). For the brewing group, 100 °C water was used and subsequently allowed to cool naturally to 40 °C. In the decocting group, water was boiled with sweet tea for 10 min and then also cooled naturally to 40 °C. A tea-to-water ratio of 1:10 was employed for both brewing and decoction to increase the content of active substances. Following brewing or decoction, the tea infusions were strained through sterile gauze to remove solid residues. The filtrates were lyophilized at −50 °C and 10 Pa for 48 h. The dry material yield was 0.842 ± 0.031 g per 100 mL of infusion for the brewing group and 1.267 ± 0.045 g per 100 mL of infusion for the decoction group (n = 3). Subsequent analyses were performed on a dry-weight basis unless otherwise stated. After the tea infusion was freeze-dried, it was deemed ready for further experiments and analysis.

#### 2.2.2. In Vitro Digestion

(1) Preparation of simulated digestive fluids

Simulated salivary digestive fluid (SSF), simulated gastric digestive fluid (SGF), and simulated intestinal digestive fluid (SIF) were prepared in accordance with the formulations specified in Supplementary Table S1.

Each digestive fluid was adjusted to a final volume of 400 mL and stored at −20 °C for subsequent use. Calcium chloride dihydrate (CaCl_2_·2HO) was added during the digestion phase.

(2) Oral digestion

Four milliliters of simulated salivary digestive fluid (SSF) was heated to 37 °C. Subsequently, 5 mL portions of the extract, calcium chloride (CaCl_2_) solution, salivary amylase, and water were added to achieve a 1-fold SSF concentration. The oral simulants were transferred to an oscillating incubator and incubated at 37 °C for 2 min.

(3) Gastric digestion

Eight milliliters of simulated gastric digestive fluid (SGF) was heated to 37 °C for 5 min. The sample, following incorporation into an oral bolus, was mixed with the preheated SGF, and the pH of the mixture was rapidly adjusted to 3.0 using 5 M hydrochloric acid (HCl). Calcium chloride (CaCl_2_) and pepsin were subsequently added. The pH of the mixture was verified and readjusted to 3.0 if necessary. The mixture was then diluted with water to achieve a 1-fold simulated salivary digestive fluid (SSF) concentration.

The resulting solution was incubated in a 37 °C shaking water bath at 100 revolutions per minute (r/min) under dark conditions for 2 h.

(4) Small-intestine digestion

Eight milliliters of simulated intestinal digestive fluid (SIF) was heated to 37 °C for 5 min. The chyme was mixed with the preheated SIF, and then calcium chloride (CaCl_2_), pancreatin, and bovine bile powder were added. The pH of the mixture was then adjusted to 7.0 using 5 M sodium hydroxide (NaOH). The resulting mixture was diluted with water to achieve a 1-fold SIF concentration and subsequently incubated in a 37 °C shaking water bath at 100 revolutions per minute (r/min) under dark conditions for 2 h. The digesta was freeze-dried for subsequent analysis.

(5) Calculation of retention rate

The retention rate (RR, %) was computed to precisely quantify bioaccessibility after each digestive phase. The following formulas were employed:

Gastric Retention (%) = (Content after Gastric Digestion/Initial Oral Content) × 100%

Intestinal Retention (%) = (Content after Intestinal Digestion/Content after Gastric Digestion) × 100%

A flowchart depicting the experimental procedures used for in vitro digestion is shown in Supplementary Figure S1.

#### 2.2.3. In Vitro Fecal Fermentation

(1) Acquisition of Intestinal Microbiota

A 10% phosphate-buffered saline (PBS) solution was prepared by dissolving phosphate salts in distilled water and adjusting the volume to 1.0 L; sterilization was then performed at 121 °C for 30 min. The phosphate-buffered saline (PBS) solution was prepared by dissolving 8.0 g of NaCl (136.9 mM), 0.2 g of KCl (2.7 mM), 1.44 g of Na_2_HPO_4_ (10.1 mM), and 0.24 g of KH_2_PO_4_ (1.8 mM) in 900 mL of distilled water. The pH was adjusted to 7.4 using 1 M HCl or NaOH, and the volume was brought to 1.0 L with additional distilled water. The solution was sterilized via autoclaving at 121 °C for 30 min and stored at 4 °C until use.

The human fecal flora model was utilized for in vitro fermentation in accordance with a previously published approach, with a few minor adjustments [15]. Six healthy volunteers (three men and three women, aged 20 to 26) who did not take supplements such as polysaccharides or polyphenols as part of their daily diets and had not been treated with probiotics or antibiotics in the preceding 90 days provided feces samples. The volunteers also underwent rigorous training in sampling procedures prior to sampling. The collected fecal samples were diluted with phosphate buffer (32%, *w*/*v*), immediately vortexed, and further centrifuged (5 min, 500 rpm/min) to remove a large quantity of food residues in the fecal suspension. Eventually, the supernatant was taken for use as the fecal slurry in subsequent experiments. The fecal inoculum was derived from a pool of six healthy donors, a sample size consistent with the INFOGEST standardized protocol and our team’s prior studies using identical methodology. This approach balances the need to capture key inter-individual microbiota variations with experimental feasibility. Pooling samples minimized idiosyncratic variations and provided a community-averaged response representative of core microbial functions.

(2) Preparation of Fermentation Medium

Preparation: In total, 4.5 g of NaCl, 4.5 g of KCl, 2.0 g pectin, 4.0 g of mucin, 0.69 g of MgSO_4_·H_2_O, 1.0 g of guar gum, 0.8 g of L-cysteine, 0.02 g of deoxygenated L-heme, 0.5 g of KH_2_PO_4_, 3.0 g of casein, 2.0 g of arabinogalactan, 1.5 g of NaHCO_3_, 0.4 g of bile salts, 0.005 g of FeSO_4_·7H_2_O, 0.08 g of CaCl_2_, 1 mL of Tween 80, and 4 mL (0.025%, *w*/*v*) of resazurin (anaerobic indicator) were dissolved in distilled water. The volume was then adjusted to 1.0 L using a volumetric flask; this step was followed by sterilization at 121 °C for 30 min. The sterilized solution was stored until subsequent use.

(3) In Vitro Fermentation

Fecal slurry and basal medium were preheated to 37 °C. In a sterile tube, 5 mL of the preheated mixture was combined with 1 g of freeze-dried tea infusion powder; this step was followed by nitrogen flushing and sealing of the tube. Basal nutrient medium without additional carbon sources served as the control. Next, 2.0 mL of 32% fecal slurry was added to the basal nutrient medium, which was placed in an Anaero Pack System (Mitsubishi Gas Chemical Co., Inc., Tokyo, Japan) and incubated at 37 °C in an anaerobic incubator (THZ-98C, Shanghai Yiheng Scientific Instrument Co., Ltd., Shanghai, China). Anaerobic fermentation of the mixture was carried out at 37 °C, with sampling at 0, 12, and 24 h for subsequent analysis.

A blank control group (Group B), given a substance containing the basal nutrient medium without any additional carbon source (i.e., no tea infusion), was included to account for the background metabolic activity of the fecal microbiota.

Participant Recruitment Criteria: The recruitment criteria for the six healthy volunteers were explicitly defined: age (20–26 years), no history of gastrointestinal diseases, no use of antibiotics/probiotics/prebiotics within the last three months, and no recent major illnesses.

Sample Anonymization and Processing: We will now describe the anonymization process in detail. Each donor was assigned a unique, non-identifiable code. To minimize inter-individual variation and further ensure anonymity, fecal samples from all six donors were immediately pooled together after collection to create a single, homogenized inoculum for all fermentation experiments. This means the reported data reflects the community-averaged response from the donor pool, and results cannot be traced back to any single individual.

All procedures performed in this study were approved by Sichuan Agricultural University Academic Ethical and Welfare Committee, and we received consent to use fecal samples from human donors; please refer to the attachment for ethical approval and Human Ethics and Consent to Participate declarations (H20250052).

A flowchart depicting the experimental procedures used for in vitro fecal fermentation is shown in Supplementary Figure S2.

#### 2.2.4. Sensory Evaluation

In sensory evaluation, the ratio of sweet tea to water was set to 1 g to 500 milliliters. The tea-to-water ratio was optimized according to analytical objectives: 1:500 for sensory evaluation to reflect typical consumption conditions, and 1:10 for chemical characterization to ensure detection sensitivity was high enough. This dual-ratio approach follows established practices in food matrix analysis, where sensory relevance and analytical detection requirements may necessitate different preparation methods.

Twelve food science majors—6 men and 6 women—aged 19–25 participated in the trial. Participants completed over 20 h of training in distinguishing the sensory differences of the sample solutions visually and quantitatively to accurately comprehend appropriate sensory evaluation terminology. Participants were trained to examine, evaluate, differentiate, and characterize the sensory qualities of the control solution and samples. Prior to the evaluation, evaluators were told to avoid performing the evaluation while hungry, to stay mentally alert, and not to eat within one hour before the evaluation. Communication between evaluators was not allowed during the evaluation. The evaluation form was scored using a 100-point scale (Supplementary Table S2). The evaluations were conducted in individual sensory booths under standardized white lighting (~500 lx). All tea infusions were served in lidded cups at a controlled temperature of 50 ± 2 °C. The description reinforced the strict pre-session guidelines for panelists and the prohibition of communication. These details are now fully integrated into this manuscript.

#### 2.2.5. Indicator Analysis

(1) Total flavonoid determination

A 1 mg/mL sample solution was prepared by mixing 10 mg of freeze-dried sample with 10 mL of ultrapure water. The total flavonoid content in sweet tea leaf samples was determined using the AlCl_3_ colorimetric method. We mixed 1.0 mL of diluted sample with 0.3 mL of 5% sodium nitrite, shook the mixture, and waited for 6 min. We then added 0.3 mL of 10% aluminum nitrate, shook the mixture again, and waited for another 6 min. Then, we added 4 mL of 5% sodium hydroxide, shook the mixture, and let it stand for 15 min before measuring the absorbance at 510 nm. Each assay batch included reagent blanks and standard curves (gallic acid/catechin, 0–100 mg/L, R^2^ > 0.995). Measurements were performed in triplicate.

(2) Total polyphenol determination

The sample’s total polyphenol content was measured using the Folin–Ciocalteu method. For reagent preparation, we prepared a 0.2 mg/mL gallic acid stock solution by dissolving 2 mg of gallic acid in ultrapure water and diluting it to 10 mL. We then prepared a 7.5% sodium carbonate solution by dissolving 3.75 g of sodium carbonate in water and diluting it to 50 mL (the resulting product will remain stable at room temperature for up to a month). For the Folin phenol reagent, we diluted 5.0 mL to 50 mL in a volumetric flask with water, preparing it fresh as needed.

(3) Analysis of the functional components

The analysis of the functional components of GB, BB, GD, and BD was carried out using high-performance liquid chromatography (LC3000, Anhui Wanyi Technology Co., Ltd., Hefei, China). System suitability was confirmed using reference standards. A pooled quality control (QC) sample was analyzed periodically throughout the sequence. Quantification was based on multi-point calibration curves (R^2^ > 0.99).

① Determination of the content of triterpenoid glycosides and geniposide

The samples were analyzed using high-performance liquid chromatography (HPLC), with three replicates per sample. Chromatographic column: target C18 (250 mm ×4.6 mm, 5 μm). Mobile phase: (A) 100% methanol; (B) 0.2% glacial acetic acid = 48:52. Flow rate: 1 mL/min. Column temperature: 30 °C. Injection volume: 20 μL. Detection wavelength: 285 nm.

② Determination of catechol content

HPLC analysis was performed on the samples, with three replicates for each. Chromatographic column: target C18 (250 mm × 4.6 mm, 5 μm). Mobile phase: (A) 100% methanol; (B) 0.2% glacial acetic acid. Flow rate: 0.9 mL/min. Column temperature: 35 °C. Injection volume: 10 μL. Detection wavelength: 278 nm.

(4) Antioxidant activity determination

① DPPH radical-scavenging ability

Ascorbic acid (Vc) was used as a positive control in each batch. Sample activities were expressed as Vc equivalents. Vc (positive control), Control, LEHs, and MRPs were dissolved in distilled water to create solutions with concentrations of 0.2 mg/mL and 0.4/0.6/0.8/1.0 mg/mL, respectively. Briefly, 0.0197 g of DPPH was combined with 100 mL of ethanol to make a 1 mM DPPH solution. This solution was diluted to a concentration of 0.70 ± 0.02 before testing. To test the samples, 2 mL of the test sample was combined with 2 mL of the DPPH solution (0.2 mM). After the DPPH solution was added, the mixture was immediately shaken and left at room temperature for 30 min in a light-free location [16]. The absorbance at 517 nm was then recorded using a spectrophotometer (UV-1800PC, Beijing Beike Hengxin Scientific Instrument Co., Ltd., Beijing, China). DPPH free-radical-scavenging activity was calculated as follows:

DPPH radical-scavenging activity (%) = $1-\frac{A_{1}-A_{2}}{A_{0}}$

A0—Absorbance at 517 nm after reaction of 2 mL distilled water with DPPH;

A1—Absorbance values of 2 mL sample solution and DPPH solution at 517 nm;

A2—Absorbance values of 2 mL anhydrous ethanol and sample solution at 517 nm.

② ABTS Radical-Scavenging Capacity

After 12 h of incubation at room temperature without light, ABTS (7 mM) was oxidized with potassium persulfate (2.45 mM) to acquire ABTS solution. Before use, the ABTS solution was freshly produced and diluted with ethanol to achieve an absorbance of 0.70 ± 0.02, as validated by spectrophotometry. To initiate the reaction, 1 mL of the sample solution was added to 4 mL of ABTS solution and mixed thoroughly. After keeping the reaction mixture at room temperature in the dark for 6 min, we measured the absorbance at 734 nm. The ABTS scavenging activity was calculated as follows:

ABTS radical-scavenging activity (%) = $1-\frac{A_{1}-A_{2}}{A_{0}}$

A0, which denotes the absorbance value at 734 nm after the reaction of 1 mL of distilled water with 4 mL of ABTS;

A1, which denotes the absorbance values of the 1 mL sample solution and 4 mL ABTS solution at 734 nm;

A2, which denotes the absorbance values of 4 mL of anhydrous ethanol and the 1 mL sample solution at 734 nm

(5) pH

Samples were taken at 0, 6, 12, and 24 h during fermentation; placed in test tubes; and cooled in an ice-water bath to stop the reaction. The pH of each sample was measured three times with a pH meter (FE28, Mettler Toledo, Zurich, Switzerland).

(6) Determination of Short-Chain Fatty Acid (SCFA) Content in Fermentation Products

The composition and content of short-chain fatty acids (SCFAs) in the fermentation products were analyzed using gas chromatography (7890A-5977B, Agilent Technologies Inc., Santa Clara, CA, USA). At 0 h, 12 h, and 24 h during the fermentation process, a 1 milliliter sample of fecal culture was taken and centrifuged, and the supernatant was collected. The supernatant was mixed with concentrated hydrochloric acid and diethyl ether. The supernatant was then collected again and mixed with concentrated hydrochloric acid, filtered, and then analyzed via GC. A standard curve was prepared using six standard substances (acetic acid, propanoic acid, butyric acid, isobutyric acid, valeric acid, and isovaleric acid), and the types and contents of SCFAs in the samples were calculated based on the curve.

(7) Intestinal Flora Analysis

Microbial diversity detection was performed for the four groups of samples that had been fermented for 24 h. DNA was extracted using the TIANamp Stool DNA Kit, and the detection analysis was carried out by Shenzhen Weikewang Technology Group Co., Ltd. (Shenzhen, China). For high-throughput sequencing, the V3-V4 region of the 16S rDNA was amplified and sequenced using Illumina Miseq. In addition, all results are based on sequencing reads and operational taxonomic units (OTUs), and Mothur was used to analyze α diversity.

#### 2.2.6. Principal Coordinate Analysis

Principal Coordinate Analysis (PCoA) is a dimensionality reduction technique used to visualize the similarity of high-dimensional data. The process begins with calculating a distance matrix that quantifies the dissimilarity between samples. This matrix is then subjected to eigenvalue decomposition to extract principal coordinates, which represent the directions of maximum variance in the data. The top principal coordinates are selected to capture the most significant patterns. The original data are projected onto these coordinates, transforming them into a lower-dimensional space. Finally, samples are plotted in two or three dimensions to analyze their distribution and clustering, revealing patterns and relationships within the data. PCoA simplifies complex datasets, aiding researchers in understanding the relationships between samples.

### 2.3. Statistical Analysis

All data are expressed as means and standard deviations. Unless specified, each experiment was replicated independently in triplicate. Statistical significance was determined using ANOVA and Tukey’s test (SPSS 22.0). Categorical variables were analyzed with Origin 2024, and OTU relative abundance differences were adjusted using Tukey’s HSD test in R. The homogeneity of variances was assessed using Levene’s test, and the normality of data distribution was checked using the Shapiro–Wilk test. For data that met the assumptions of normality and homogeneity of variance, one-way analysis of variance (ANOVA) was used, followed by Tukey’s honestly significant difference (HSD) post hoc test for multiple comparisons. For data that did not meet the homogeneity-of-variance assumption, a transformation was applied prior to ANOVA. Samples were randomized across different batches and analysis sequences to avoid systematic bias. Only data from analytical batches passing QC checks (e.g., calibration R^2^ > 0.99, replicate RSD < 5%) were included in the final statistical analysis.

## 3. Results and Discussion

### 3.1. Comparison of Active Components and Sensory Scores Among Four Types of Tea Infusions

As shown in Table 1, differences in active ingredients were found in the sweet tea processed with various fermentation and brewing methods. The leaves prepared using the GD method had the highest polyphenol and flavonoid content, while the leaves prepared using the BB method had the lowest. Catechin content was lower than dihydrochalcone content. As shown in Table 2, the sensory differences between the tea infusions were notable: BB had the highest overall score, while GD had the lowest. The overall score was positively correlated with the sweetness score and negatively correlated with the bitterness and astringency scores. There was no significant difference in sourness among the four groups of samples. The reason for the sensory difference may be that the dihydrochalcones in sweet tea introduce sweetness [17], while catechin substances produce bitterness and astringency [18]. The greater bitterness and astringency of GD lower its overall taste score compared to BD, despite GD’s greater sweetness. This indicates that increased bitterness and astringency significantly reduce overall taste preference, leading to a greater consumer preference for BB and BD.

Brewing exhibited a lower extraction efficiency compared to decoction, primarily due to the inability to maintain a consistent water temperature. This temperature variation resulted in distinct compositional profiles between the brewing and decoction groups. High temperatures reduce tea’s fresh taste and can cause oxidative degradation of tea polyphenols [19], causing the tea infusion to turn yellow, and a low brewing water temperature makes it difficult for the internal substances to extract, resulting in a light infusion color and a milder taste. A recent comprehensive investigation by Jin [20] demonstrated that brewing conditions, particularly temperature and time, induce significant metabolic profile changes in green tea, which directly underpin its sensory properties. Our results substantiate this through the specific impact of temperature and time. Furthermore, the gradation in susceptibility observed across tea types—from green tea (the most) to black tea (the least)—aligns with the extent of fermentation and associated biochemical stability. This gradation echoes the findings of Cao [21], who reported that the polymerization of catechins into more stable compounds like theaflavins and thearubigins during fermentation reduces interaction between leaf constituents and brewing water minerals.

The significant differences in sensory attributes (e.g., the greater bitterness/astringency of the GD-prepared tea) observed among the tea infusions prepared using different methods (brewing vs. decoction) can primarily be attributed to the variations in the extraction and degradation of polyphenols and flavonoids, as discussed above. Importantly, these processing methods involve distinct thermal profiles, which may also differentially influence the evolution of aroma compounds [22]. For instance, Zhai reported that thermal energy during brewing is a key driver of the formation of dimethyl sulfide (DMS) from its precursor [23]. Thus, the sustained boiling in decoction likely promotes the formation or degradation of specific volatile compounds like DMS to a different extent compared to brewing, potentially contributing to the observed differences in the overall flavor perception and acceptability. The complex interplay between taste and aroma chemistry under varying processing conditions merits further investigation.

### 3.2. Comparison of Active Components and Antioxidant Activities in Four Types of Tea Infusions

The high stability of polyphenols and flavonoids in the salivary phase (retention > 98%) aligns with the findings of Xie [24], who reported >95% retention for multiple flavonoid types during oral digestion, confirming the limited enzymatic susceptibility of these compounds to salivary α-amylase.

**Figure 1:**
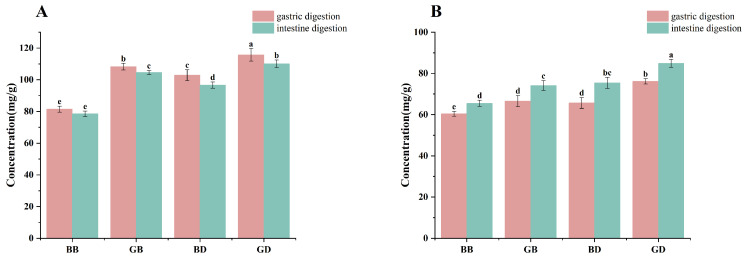
Changes in the content of active components in tea infusions after in vitro simulated gastrointestinal digestion. GB, green-tea brewing group; BB, black-tea brewing group; GD, green-tea decoction group; BD, black-tea decoction group. The changes in content of polyphenols (**A**), and flavonoids (**B**) during the digestion process (gastric digestion and intestine digestion). Lowercase letters (^a–e^): significant differences within different samples (*p* < 0.05).

As illustrated in Figure 1A, polyphenol content declined during in vitro simulated digestion across all tea infusion groups (BB, GB, BD, and GD). This reduction may be attributed to the hydrolysis of phenolic compounds under simulated gastric and intestinal conditions. Gastrointestinal digestive enzymes break down proteins, creating new peptide segments that also bind with phenolic compounds, further decreasing their content in gastric juice [25]. It is worth noting that phenolic content was higher in the simulated gastric phase (pH ≈ 2–3) than in the intestinal phase (pH ≈ 6–7). This may be due to acid hydrolysis of bound phenolics under strong acidity, which releases free, detectable compounds. In contrast, under near-neutral conditions, phenolics may become less soluble, interact with digestive components, or undergo degradation, leading to lower detectable levels. Similar trends have been reported in previous studies on polyphenol bioaccessibility [26]. Furthermore, the gastrointestinal environment, with harsh pH, dissolved oxygen, and metabolic enzymes, leads to rapid degradation or metabolism of polyphenols, hindering their stabilization [27].

Flavonoid content first decreased and then increased, basically returning to the level before digestion (shown in Figure 1B). This is because flavonoids can undergo acid hydrolysis or be converted into other compounds, meaning they are degraded to varying degrees in the digestive tract [28]. It may also be that some flavonoid substances bind with digestive enzymes, affecting the position and number of hydroxyl groups on flavonoid compounds, thereby reducing the activity of flavonoid substances. Yu et al. [10] indicated that the five flavonoid compounds mainly bind to pepsin through hydrophobic interactions as well as hydrogen bonds and electrostatic forces. The enzymes in the intestinal fluid digestion group may act on bound phenolic substances, and the digestive action of intestinal enzymes causes flavonoid substances to be released from the matrix. At the same time, flavonoid substances that are abundant in sweet tea, such as dihydrochalcones and quercetin, can exist stably in neutral or slightly alkaline intestinal fluid media [29].

**Figure 2:**
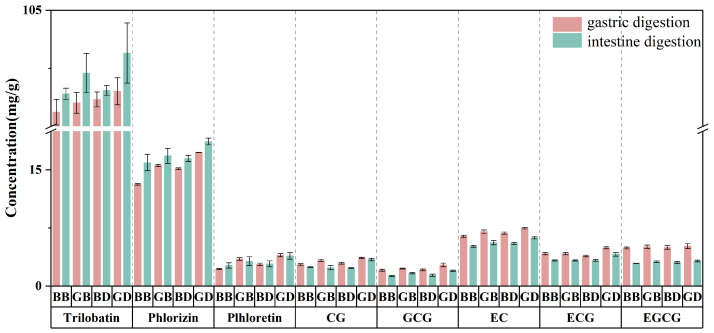
The changes in the content of dihydrochalcones and catechins during the digestion process (gastric digestion and intestine digestion). EC, epicatechin; CG, catechin gallate; ECG, epicatechin gallate; GCG, gallocatechin gallate; EGCG, epigallocatechin gallate.

**Figure 3:**
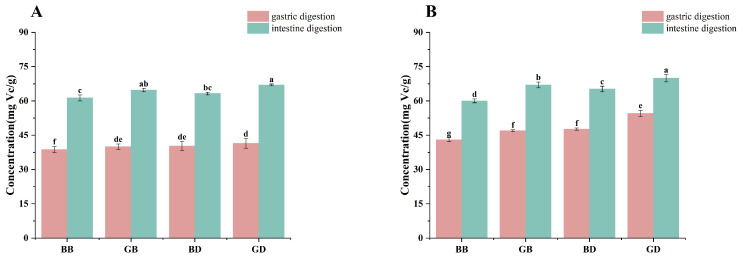
Changes in antioxidant activity of tea infusions prepared using different processing methods during in vitro digestion. Results were expressed as mg equivalents of Vc per digestive juice (mg Vc/g digestive juice). GB, green-tea brewing group; BB, black-tea brewing group; GD, green-tea decoction group; BD, black-tea decoction group. (**A**) ABTS radical-scavenging activity—Vc (1 mg/mL) was used as standard. (**B**) DPPH radical-scavenging activity—Vc (1 mg/mL) was used as standard. Lowercase letters (^a–g^): Significant differences within different samples (*p* < 0.05).

As shown in Figure 2, the content of catechins, including C, CG, GCG, EC, ECG, and EGCG, decreased after gastrointestinal digestion. EGCG exhibited the most significant reduction, which was attributable to the auto-oxidative degradation of its ortho-pyrogallol moieties on the B-ring in the neutral intestinal environment [30]. Conversely, catechin (C) fell below the detection limit, likely due to epimerization and interconversion under the alkaline conditions of the intestinal fluid, which may also contribute to the relative stability of epicatechin (EC) [31]. Critically, the initial differences in catechin profiles among the four tea infusion groups (GB, BB, GD, and BD), as established in Table 1, were largely preserved in the final digestate (Figure 2). This indicates that the compositional outcome of digestion is predominantly determined by the initial composition of the tea infusion. The degradation and potential interconversion of catechins directly influenced the dynamic changes in antioxidant activity. The intestinal phase showed a recovery in antioxidant capacity, which paralleled the trend observed in flavonoid content (Figure 1B). Notably, trilobatin, the predominant dihydrochalcone in sweet-tea infusions (Table 1), exhibited significant differences among the groups after digestion (Figure 2), with its content following the order GD > GB > BD > BB. This pattern strongly suggests that trilobatin is a key contributor to the antioxidant activity, explaining why the GD group, with the highest retained dihydrochalcone content, demonstrated superior ABTS and DPPH radical-scavenging activities in the intestinal phase (Figure 3).

The bioaccessibility analysis revealed that polyphenols and flavonoids exhibit good tolerance to the acidic gastric environment, with retention rates for polyphenols and flavonoids falling within the ranges of 81.9–88.2% and 83.3–89.6%, respectively. Meanwhile, exceptional stability was observed in the intestinal phase, as evidenced by the retention rates of 94.2–95.5% for polyphenols and 106.7–121.9% for flavonoids (as shown in Table S3). This indicates negligible degradation and suggests potential conversion or release of these compounds.

Trilobatin exhibits moderate enzymatic stability through static-quenching interactions with digestive enzymes. It binds to pepsin via hydrogen bonds/van der Waals forces and to trypsin mainly through hydrophobic interactions, inducing conformational changes in both enzymes while maintaining structural integrity. Metal ions reduce binding affinity, but pH-specific stability suggests resilience during digestion [32].

Phloretin maintains gastrointestinal stability through reversible enzyme binding and structural modifications. It forms non-covalent complexes with digestive enzymes via static quenching driven by hydrogen bonding and van der Waals forces, inducing conformational changes in pepsin while preserving its own scaffold. This strategy maintains antioxidant capacity while optimizing bioavailability, and pH-dependent stability ensures gastric integrity (pH 2.0) with controlled intestinal release [33].

During digestion, changes in ABTS (Figure 3A) and DPPH (Figure 3B) antioxidant activities paralleled those in flavonoid content. The intestinal digestion phase exhibited an upward trend in antioxidant activity, likely driven by flavonoid release. Due to pepsin, the ABTS radical-scavenging capacity of herbal extracts decreased significantly in gastric fluid [34]. After intestinal digestion, this capacity was restored to pre-digestion levels, indicating pepsin’s role in modulating scavenging activity during gastric digestion. Trilobatin, the primary flavonoid component in the sweet tea infusion (Table 1), exhibited significant post-digestion differences across the BB, BD, GB, and GD groups (Figure 2), with the green tea groups exceeding the black tea groups, and the decocted groups surpassing the brewed groups, as confirmed by both our data and Cao [22]. These results suggest trilobatin is a key determinant of antioxidant activity. Unfermented tea retains more dihydrochalcone compounds, and the decocting process further enhances their release. Consequently, in intestinal digestive fluid, the GD group exhibited higher dihydrochalcone content than the BB, GB, and BD groups, explaining the superior ABTS and DPPH radical-scavenging activities in GD.

### 3.3. Analysis of Short-Chain Fatty Acid Content and Microbiota in Four Types of Tea Infusion Fermentation Liquids

**Figure 4:**
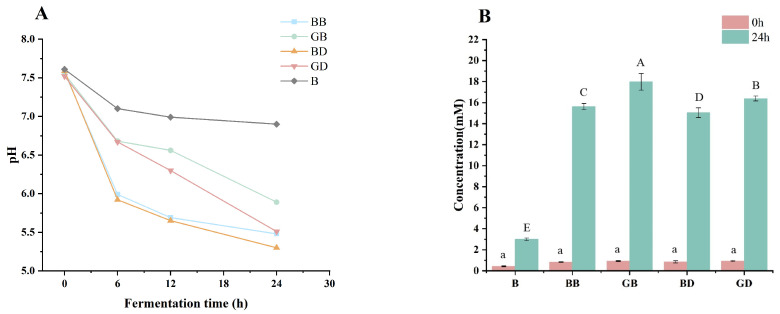
Changes in pH value (**A**) and SCFAs (**B**) in tea broth fermentation solution. GB, green-tea brewing group; BB, black-tea brewing group; GD, green-tea decoction group; BD, black-tea decoction group; B, fermentation-in vitro-without-the-sample group. Lowercase letters (^a^): significant differences within different samples in 0 h (*p* < 0.05). Uppercase letters (^A–E^): significant differences within different samples in 24 h (*p* < 0.05).

As displayed in Figure 4A, the pH values of all the groups decreased, as polyphenols and flavonoids can modify the production of SCFAs by changing the composition and thus the function of gut microbiota, leading to a decrease in the pH value of the fermentation culture. The decrease in pH during fermentation is primarily attributable to the accumulation of short-chain fatty acids (SCFAs), produced by gut microbiota. While polyphenols and flavonoids do not directly lower pH, they modulate microbial composition by promoting the growth of SCFA-producing bacteria, which metabolize these compounds into SCFAs. The accumulation of SCFAs, such as acetic, propionic, and butyric acids, leads to a decrease in pH within the fermentation system [35]. As shown in Figure 4B, the distinct pH values among the sample groups during fermentation indicated a correlation between pH and short-chain fatty acid (SCFA) production during in vitro fermentation. Total SCFA content in all the sample groups increased significantly compared to the B group at both 0 h and 24 h. Notably, BD exhibited the highest final SCFA content, whereas GB had the lowest. The health benefits of plant polyphenols are largely mediated by gut microbiota, with their efficacy influenced by factors such as polyphenol type, plant source, gut microbiome composition, and intestinal retention time [36]. Thus, the observed disparities in SCFA levels may stem from differences in polyphenol types.

**Figure 5:**
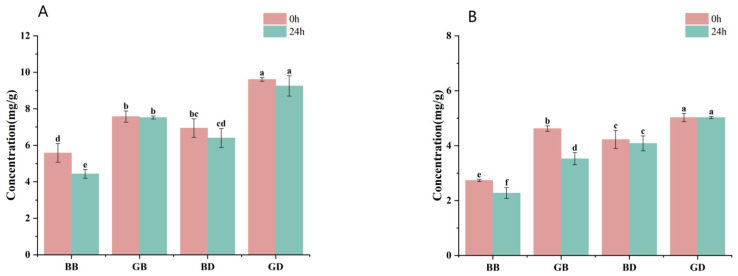
Changes in polyphenols (**A**) and flavonoids (**B**) in tea broth fermentation solution. GB, green-tea brewing group; BB, black-tea brewing group; GD, green-tea decoction group; BD, black-tea decoction group. Lowercase letters (^a–f^): significant differences within different samples (*p* < 0.05).

As shown in Figure 5A,B, following in vitro simulated fecal fermentation, GD maintained stable polyphenol and flavonoid levels, whereas BB polyphenol levels decreased by 20.61% and levels of GB flavonoids decreased by 23.81%. These changes may arise from two factors: progressive acidification during fermentation, which promotes phenolic degradation under acidic conditions, and phenolic conversion. Studies suggest polyphenols’ limited bioavailability (incomplete intestinal absorption, low target-organ delivery, and rapid metabolism) reduces their bioactivity, though biotransformation in the gut enhances it [37]. Specifically, colonic polyphenols are extensively metabolized by gut microbiota into low-molecular-weight phenolic acids [38]. Flavan-3-ols, such as epigallocatechin and epicatechin, undergo C-ring cleavage, resulting in small phenolic metabolites, while flavonols, flavanones, and other classes follow similar pathways [39,40]. Dihydrochalcones like neohesperidin are converted into 3-(3-hydroxy-4-methoxyphenyl)propionic acid derivatives [41]. Tea polyphenols (catechins and theaflavins) exert antimicrobial/anticancer effects via hydroxyl–lipid bilayer hydrogen bonding, and phenolics inhibit bacteria by binding enterotoxins [42,43]. Collectively, these processes drive phenolic content changes.

**Figure 6:**
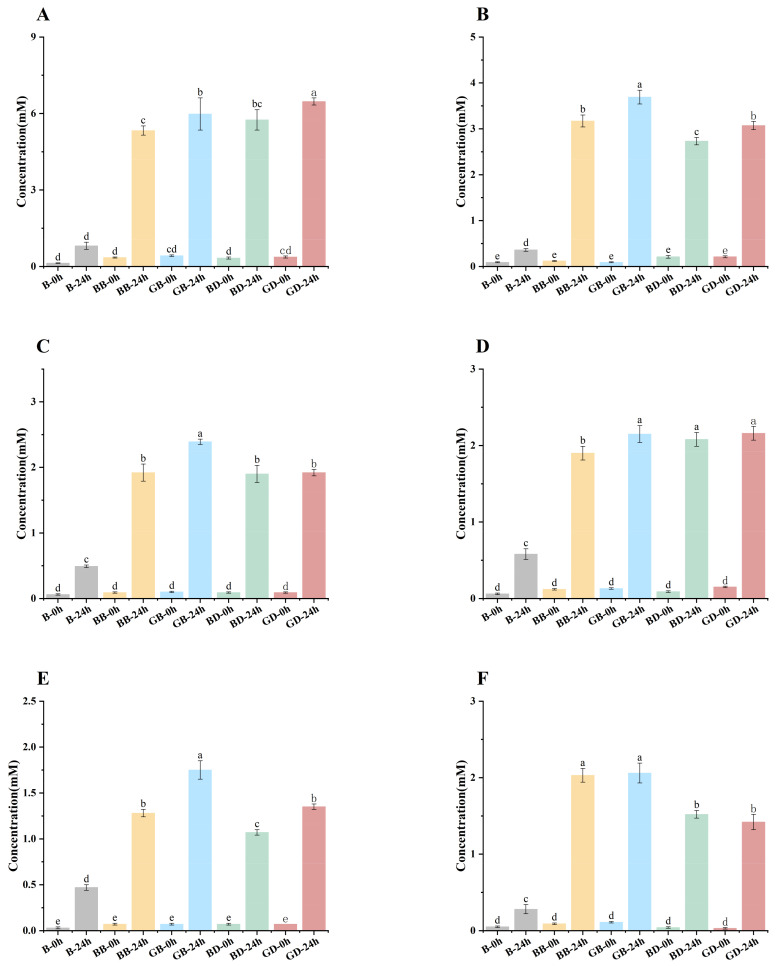
Content of each component of SCFAs. GB-0 h (24 h), green-tea brewing group at 0 h (24 h); BB-0 h (24 h), black-tea brewing group at 0 h (24 h); GD-0 h (24 h), green-tea decoction group at 0 h (24 h); BD-0 h (24 h), black-tea decoction group at 0 h (24 h); B-0 h (24 h), fermentation-in vitro-without-the-sample group at 0 h (24 h). Changes in acetic acid (**A**), propanoic acid (**B**), butyric acid (**C**), isobutyric acid (**D**), valeric acid (**E**), and isovaleric acid (**F**). Lowercase letters (^a–e^): significant differences between different samples (*p* < 0.05).

SCFAs—including acetic, propanoic, butyric, isobutyric, valeric, and isovaleric acid—are produced by gut microbiota, influenced by diet and flora composition, and critical for intestinal health. As shown in Figure 6A–F, taking BD-24 as an example, during fermentation, BD-24 exhibited increased levels of acetic, propionic, and butyric acid. Correlations between key SCFAs (acetic, propionic, and butyric acid) and polyphenols (e.g., epicatechin and protocatechuic acid) suggest polyphenols promote SCFA production [44]. Similarly, phlorizin intake elevated fecal levels of acetic, propionic, and butyric acid but not isobutyric, valeric, or isovaleric acid. Sweet-tea-derived trilobatin also increases levels of SCFAs, particularly butyric acid, in mice [45].

Finally, in all fermentation processes, the contents of isobutyric acid, valeric acid, and isovaleric acid were low, as shown in Figure 5. Similarly, phlorizin intake elevated fecal levels of acetic, propionic, and butyric acid but not isobutyric, valeric, or isovaleric acid [46].

Notably, the black-tea group exhibited higher SCFA content than the green-tea group. This disparity has been associated with the inhibitory effects of black-tea extract on pancreatic α-amylase, which increases cecal starch availability for SCFA production [47]. Brewing and decocting minimally modulated SCFA composition, likely due to interactions between polyphenols, flavonoids, and gut microbiota being governed by their content and composition.

The microbiome significantly modulates human health, especially in nutrient acquisition, energy metabolism, and immune system development. The human gut, the body’s largest microbial reservoir, contains around 400 bacterial species. We identified 971,075 raw sequencing reads, of which 933,502 were high-quality gene sequences with an average length of 412 bp per sample. Rarefaction curves were used to show species richness and evaluate sequencing depth. When these curves level off, it suggests that the sequencing data is adequate for capturing the microbial diversity in the samples. As can be seen in Figure S3, as sampling depth increases, the number of OTUs detected rises but eventually levels off, suggesting that most species have already been identified. This data is suitable for statistical analysis and reliable.

**Figure 7:**
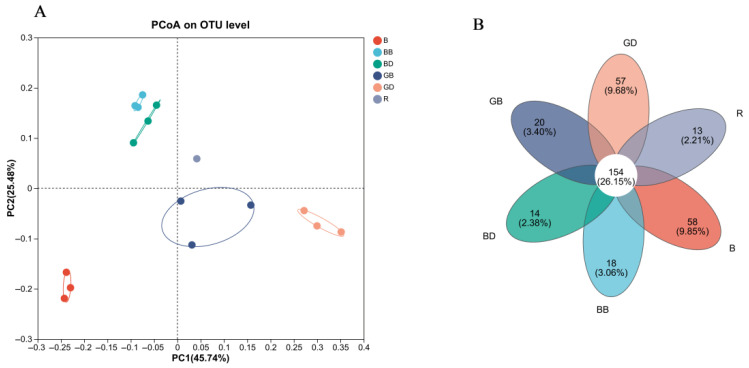
PCoA analysis and Venn diagram. GB, green-tea brewing group at 24 h; BB, black-tea brewing group at 24 h; GD, green-tea decoction group at 24 h; BD, black-tea decoction group at 24 h; B, fermentation-in vitro-without-the-sample group at 24 h; R, raw fecal sample group at 24 h. (**A**) Principal co-ordinate analysis (PCoA) is a non-constrained data reduction analysis method used to study similarities or differences in sample community composition. (**B**) Venn diagrams focus on analyzing and displaying the unique and shared species numbers among different groups, with the graphical representation being a petal diagram. The petals represent the number of species unique to each corresponding group, and the center shows the number of species shared by all groups.

PCoA (Figure 7A) demonstrated that sweet-tea polyphenols and flavonoids significantly modulated gut microbiota composition. Figure 7B reveals 154 shared operational taxonomic units (OTUs) among groups following sweet-tea-infusion addition, with unique species present in each group, indicating enhanced species richness and diversity.

**Figure 8:**
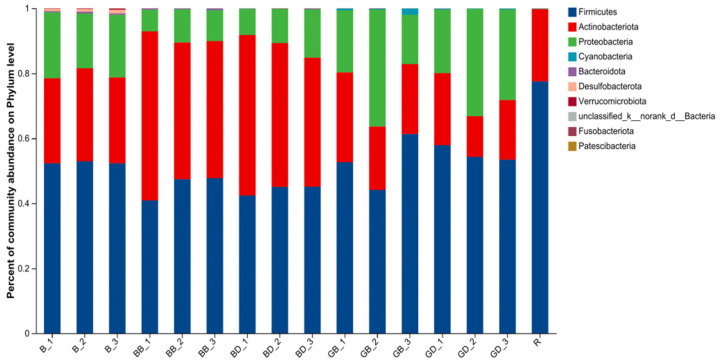
The composition and proportion of the top N species in terms of abundance across all samples. GB, green-tea brewing group at 24 h; BB, black-tea brewing group at 24 h; GD, green-tea decoction group at 24 h; BD, black-tea decoction group at 24 h; B, fermentation-in vitro-without-the-sample group at 24 h; R, raw fecal sample group at 24 h.

At the phylum level (Figure 8), the dominant microbiota included Firmicutes, Bacteroidetes, Proteobacteria, and Actinobacteria. Phenolic-rich foods typically reduce the Firmicutes/Bacteroidetes ratio, which correlates with improved gut health; a Firmicutes-dominated flora is linked to metabolic disorders like diabetes and obesity [48]. Sweet tea reduces the abundance of Proteobacteria, a phylum normally low in healthy gut microbiota. An increase in Proteobacteria is associated with microbiota imbalance, as seen in colitis and inflammatory bowel disease, potentially due to their interaction with colon cells via secretion systems [49].

**Figure 9:**
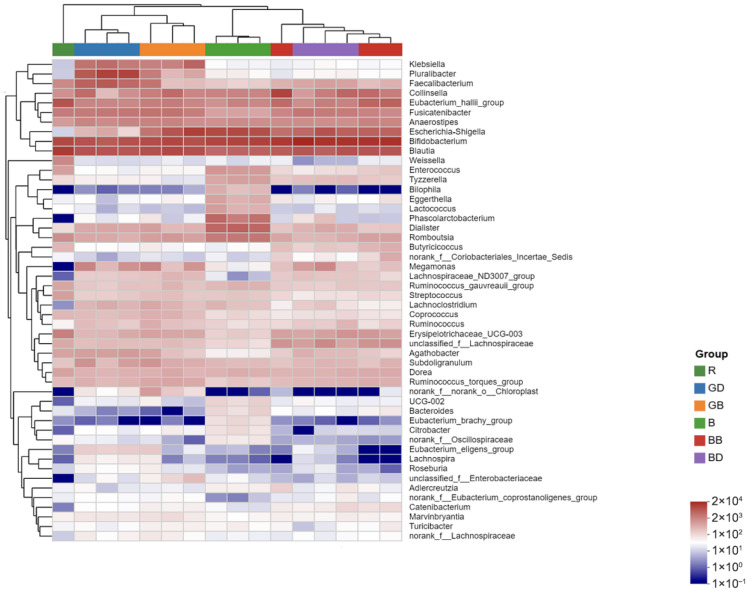
The distribution of the top dominant species in different samples/groups. GB, green-tea brewing group at 24 h; BB, black-tea brewing group at 24 h; GD, green-tea decoction group at 24 h; BD, black-tea decoction group at 24 h; B, fermentation-in vitro-without-the-sample group at 24 h; R, raw fecal sample group at 24 h.

At the genus level (Figure 9), 24 h fermentation revealed significant differences between the sample and control groups. The sample group exhibited increased abundances of Roseburia, Coprococcus, and Ruminococcus.

The observed stability of specific sweet-tea polyphenols during gastrointestinal digestion and their subsequent promotion of distinct SCFA profiles during fermentation provide a mechanistic basis for these microbial changes. The relative resistance of certain dihydrochalcones (e.g., trilobatin) to digestive degradation suggests their potential to reach the colon in a bioactive form, where they serve as substrates for microbial fermentation. This colonic metabolism directly links to the elevated production of SCFAs, particularly acetate and propionate, observed in our fermentation model.

These changes align with the findings of Zhang et al.’s [46] and Liao et al.’s [50] research on pectin–gut microbiota interactions and are physiologically significant. Roseburiaand Coprococcus, which showed increased abundance, is a known producer of butyrate and acetic acid. Physiologically, acetate serves as a crucial energy source for colonic epithelial cells and can influence peripheral cholesterol metabolism upon systemic absorption, while propionate participates in hepatic gluconeogenesis and appetite regulation. Concurrently, Ruminococcus contributes to maintaining intestinal barrier integrity, Roseburia helps mitigate inflammation, and Coprococcus may suppress immune overreactions [51,52,53].

The synergy between polyphenol stability and microbial metabolic activity underscores a prebiotic-like effect. This effect, manifested through the increased abundance of SCFA-producing genera coupled with elevated SCFA concentrations, likely contributes to maintaining gut barrier integrity, modulating immune responses, and producing metabolically active molecules. Thus, the in vitro patterns described here provide a plausible mechanistic basis for the potential health benefits of sweet-tea consumption, primarily mediated through the gut–microbiota–SCFA axis, demonstrating how sweet tea enhances gut health by promoting beneficial bacteria through its polyphenol and flavonoid constituents.

An elevated abundance of Klebsiella was detected in specific sweet-tea subgroups (e.g., green-tea sweet tea) relative to the untreated fecal control (Group R). While Group R (a pre-fermentation baseline) already contained Bifidobacterium and Eubacterium (key beneficial taxa), sweet-tea fermentation selectively modulated the microbiota. Most of the tea infusion groups had significantly enriched abundances of Bifidobacterium and Eubacterium relative to R, aligning with their prebiotic role. However, certain tea infusions may transiently favor Klebsiella due to tea-specific components altering redox/substrate conditions [54]. Importantly, quantities of Bifidobacterium and Eubacterium remained significantly higher than in R across most groups (*p* < 0.05), indicating dominant prebiotic effects.

Overall, brewing and decocting do not significantly modulate microbiota composition. Polyphenols and flavonoids influence gut microbiota based on content, composition, and complex factors. Sweet tea reduces quantities of Faecalibacterium, Bacteroides, and Coprococcus, aligning with studies by Most and Sun indicating that its polyphenols and metabolites promote gut health by boosting beneficial bacteria and suppressing pathogens [55,56].

## 4. Conclusions

The processing methods significantly reduced catechin content, thereby lowering astringency while preserving sweetness. Following digestion, polyphenol content decreased, whereas flavonoid levels recovered to pre-digestion values. Changes in antioxidant capacity closely paralleled trends in flavonoid content. Fermentation for 24 h lowers pH values and boosts levels of short-chain fatty acids like acetic and propionic acid, with increased abundance of beneficial bacteria such as Bifidobacterium and Eubacterium, linked to the synergistic effects of polyphenols and flavonoids in sweet tea. In vitro experiments demonstrated these compounds can regulate intestinal microbiota, suggesting potential as functional prebiotics. This study compares sweet tea and its fermented form after brewing and decoction in terms of sensory, digestive, and prebiotic aspects, guiding consumer choice and potentially expanding sweet tea consumption. Future work should clarify the mechanisms of the effects of tea components on Klebsiella and validate impacts in vivo. These findings highlight tea-type-specific microbiota regulation, with core probiotics (Bifidobacterium and Eubacterium) as primary targets.

## Figures and Tables

**Table 1:** Content of active components in four types of tea infusions (*p* < 0.05).

Concentration (mg/g)	BB	GB	BD	GD
Polyphenol	93.12 ± 2.20 ^d^	129.68 ± 0.95 ^b^	121.64 ± 1.47 ^c^	144.51 ± 1.12 ^a^
Flavone	67.08 ± 0.41 ^d^	78.32 ± 0.81 ^b^	75.88 ± 0.36 ^c^	88.97 ± 0.77 ^a^
Trilobatin	69.30 ± 0.57 ^d^	83.92 ± 1.70 ^b^	76.02 ± 0.80 ^c^	87.46 ± 0.73 ^a^
Phlorizin	12.95 ± 0.97 ^d^	15.36 ± 1.00 ^ab^	14.84 ± 1.38 ^bc^	17.30 ± 0.82 ^a^
Phloretin	1.51 ± 0.18 ^c^	2.45 ± 0.12 ^b^	2.29 ± 0.26 ^b^	2.87 ± 0.16 ^a^
C	n.d.	0.97 ± 0.04 ^a^	n.d.	0.99 ± 0.03 ^a^
CG	2.98 ± 0.01 ^d^	3.19 ± 0.01 ^b^	3.09 ± 0.02 ^c^	3.26 ± 0.01 ^a^
GCG	2.11 ± 0.13 ^c^	2.81 ± 0.09 ^a^	2.29 ± 0.01 ^b^	2.92 ± 0.03 ^a^
EC	6.74 ± 0.02 ^c^	8.05 ± 0.14 ^a^	7.33 ± 0.02 ^b^	8.26 ± 0.20 ^a^
ECG	3.56 ± 0.01 ^d^	4.36 ± 0.06 ^b^	3.76 ± 0.03 ^c^	4.90 ± 0.05 ^a^
EGCG	4.81 ± 0.01 ^c^	5.10 ± 0.02 ^a^	4.97 ± 0.09 ^b^	5.17 ± 0.02 ^a^

Each value is expressed as the mean ± SD (n = 3). Means with different letters within a line are significantly different (*p* < 0.05). n.d.: not detected. GB, green-tea brewing group; BB, black-tea brewing group; GD, green-tea decoction group; BD, black-tea decoction group; C, catechin; EC, epicatechin; CG, catechin gallate; ECG, epicatechin gallate; GCG, gallocatechin gallate; EGCG, epigallocatechin gallate. Lowercase letters (^a–d^): significant differences within rows (*p* < 0.05).

**Table 2:** Sensory evaluation scores of four types of tea infusions (*p* < 0.1).

Sensory Score	BB	GB	BD	GD
Overall	78.89 ± 12.94 ^a^	73.33 ± 10.61 ^ab^	78.33 ± 8.66 ^a^	65.00 ± 14.79 ^b^
Sweetness	59.44 ± 5.27 ^b^	51.11 ± 7.41 ^c^	45.56 ± 3.91 ^a^	55.28 ± 9.48 ^d^
Sourness	2.22 ± 2.64 ^a^	2.78 ± 3.63 ^a^	2.78 ± 2.64 ^a^	3.33 ± 3.54 ^a^
Bitterness	11.67 ± 3.54 ^c^	17.78 ± 4.41 ^b^	15.00 ± 4.33 ^bc^	22.78 ± 5.65 ^a^
Astringency	20.56 ± 6.82 ^b^	26.67 ± 5.59 ^b^	24.44 ± 3.91 ^bc^	36.67 ± 6.61 ^a^

Each value is expressed as the mean ± SD (n = 3). Means with different letters within a line are significantly different (*p* < 0.05). GB, green-tea brewing group; BB, black-tea brewing group; GD, green-tea decoction group; BD, black-tea decoction group. Lowercase letters (^a–d^): Significant differences within rows (*p* < 0.05).

## Data Availability

The original contributions presented in this study are included in the article. Further inquiries can be directed to the corresponding author.

## Supplementary Materials

The following supporting information can be downloaded at https://www.mdpi.com/article/10.3390/foods15010110/s1. Table S1: Amount of electrolyte solution used; Table S2: Sensory evaluation; Table S3: The retention rate after each digestive phase; Figure S1: Flowchart of experimental operations for in vitro digestion; Figure S2: Flowchart of experimental operations for in vitro fecal fermentation; Figure S3: Chaos curve.

## Abbreviations

The following abbreviations are used in this manuscript:

GB	Green-tea brewing group
BB	Black-tea brewing group
GD	Green-tea decoction group
BD	Black-tea decoction group
