# Supplementary material for: Multidimensional Profiling of Chinese Sweet Tea (Lithocarpus litseifolius): Processing Methods Modulate Sensory Properties, Bioaccessibility and Prebiotic Potential via Gut Microbiota Regulation
Source: Foods. 2025 Dec 30;15(1):110. doi: 10.3390/foods15010110 (PMC12785535; doi:10.3390/foods15010110)
Supplement: Supplementary file 1 [file foods-15-00110-s001.zip › foods-4007756-supplementary.pdf]

Supplementary data

Tables

Table S1 Amount of Electrolyte Solution Used

Electrolyte	Concentration	SSF（pH 7）		SGF（pH 3）		SIF（pH 7）	
		Add quantity	Final concentration	Add quantity	Final concentration	Add quantity	Final concentration
	mol/L	mL	mmol/L	mL	mmol/L	mL	mmol/L
KCl	0.5	30.2	30.2	13.8	13.8	13.6	13.6
KH_2PO_4	0.5	7.4	7.4	1.8	1.8	1.6	1.6
$NaHCO_3$	1	13.6	27.2	25	50	85	170
NaCl	2	-	-	23.6	94.4	19.2	76.8
$MgCl_2\cdot 6H_2O$	0.15	1.0	0.3	0.8	0.24	2.2	0.66
$(NH_4)_2CO_3$	0.5	0.12	0.12	1.0	1.0	-	-
HCl	6	0.18	2.2	2.6	31.2	1.4	16.8
$CaCl_2(H2O)_2$	0.3	0.05	3.0	0.01	0.3	0.08	1.2

SSF:Simulated salivary digestive fluid, SGF: simulated gastric digestive fluid, SIF: simulated intestinal digestive fluid.

Table S2 Sensory Evaluation

Sensory evaluation	Scoring criteria	Score
Overall score (personal preference)	Like	76~100
	Prefer	51~75
	Less prefer	26~50
	Dislike	＜25
Sweetness	Sweet	76~100
	Sweeter	51~75
	Slightly sweet	26~50
	Not sweet	＜25
Sourness	Sour	76~100
	More sour	51~75

	Slightly sour	26~50
	Not sour	＜25
Bitterness	Bitter	76~100
	More bitter	51~75
	Slightly bitter	26~50
	Not bitter	＜25
Astringency	Astringent	76~100
	More astringent	51~75
	Slightly astringent	26~50
	Not astringent	＜25

Table S3 The retention rate after each digestive phase

Samples	Polyphenol		Flavone	
	Gastric Retention (%)	Intestinal Retention (%)	Gastric Retention (%)	Intestinal Retention (%)
BB	88.17±3.40	95.12±3.56	89.55±5.83	106.67±3.56
GB	85.27±2.10	95.45±4.87	83.33±2.01	113.85±4.21
BD	85.12±1.09	94.17±4.24	85.33±3.48	121.88±5.21
GD	81.94±2.03	94.92±3.79	88.64±3.21	108.97±4.39

GB, green tea brewing group; BB, black tea brewing group; GD, green tea decocting group; BD, black tea decocting group.

Figures

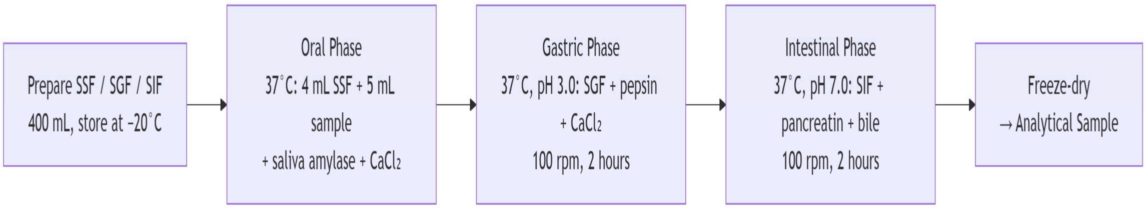

Fig S1 Flowchart of Experimental Operations for In Vitro Digestion.

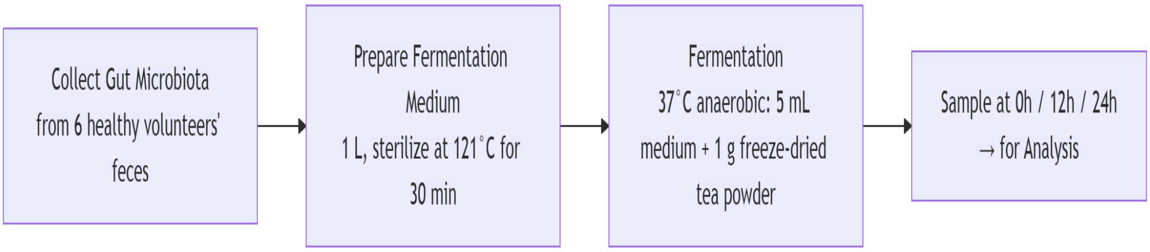

Fig S2 Flowchart of Experimental Operations for In Vitro Fecal Fermentation.

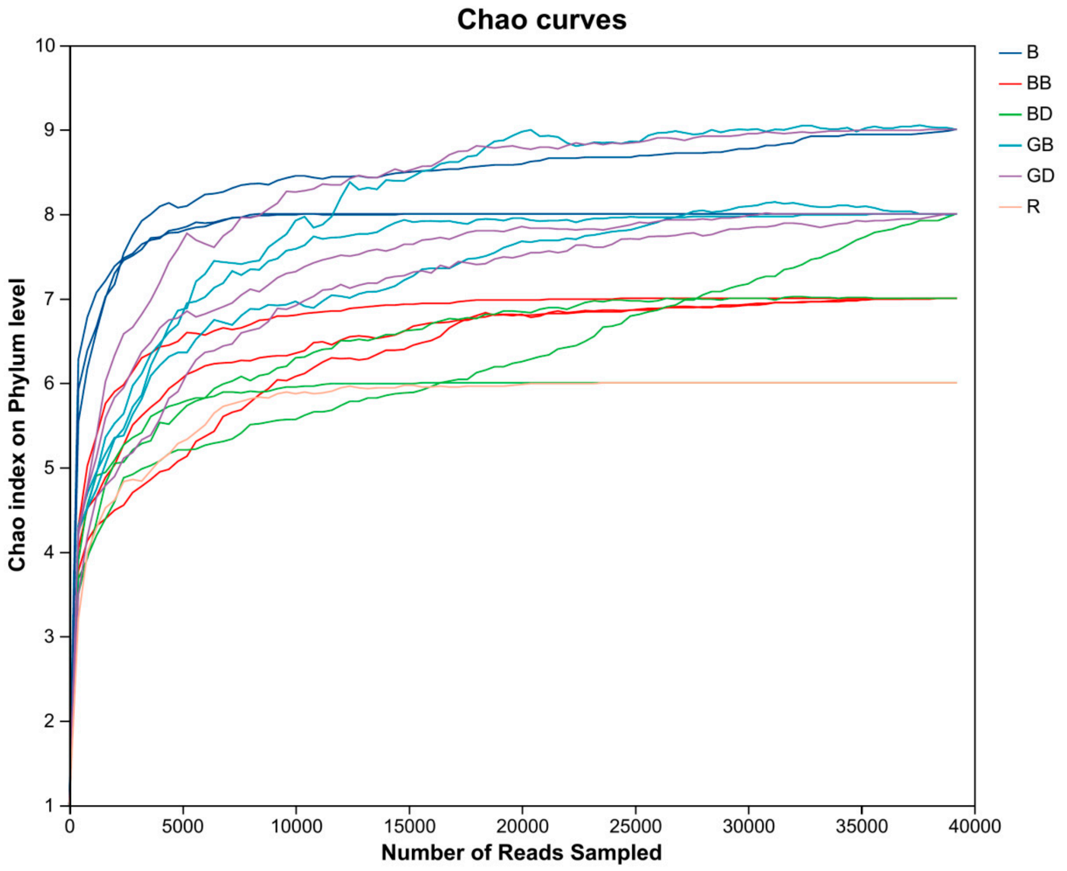

Fig S3 Chao curve. GB, green tea brewing group at 24h; BB, black tea brewing group at 24h; GD, green tea decocting group at 24h; BD, black tea decocting group at 24h; B, fermentation in vitro without the sample group at 24h; R, raw fecal sample group at 24h.
